# Robust quantitative assessment of collagen fibers with picrosirius red stain and linearly polarized light as demonstrated on atherosclerotic plaque samples

**DOI:** 10.1371/journal.pone.0248068

**Published:** 2021-03-18

**Authors:** Cherry Greiner, Stephanie Grainger, Samantha Farrow, Alena Davis, Jimmy L. Su, Matthew D. Saybolt, Robert Wilensky, Sean Madden, Stephen T. Sum

**Affiliations:** 1 Infraredx, Bedford, Massachusetts, United States of America; 2 Cytiva, Marlborough, Massachusetts, United States of America; 3 Philips Healthcare, Cambridge, Massachusetts, United States of America; 4 Hackensack Meridian Jersey Shore University Medical Center, Neptune, New Jersey, United States of America; 5 Perelman School of Medicine, University of Pennsylvania, Philadelphia, Pennsylvania, United States of America; 6 Lumicell, Newton, Massachusetts, United States of America; Monash University, AUSTRALIA

## Abstract

Collagen is an important component in maintaining structural integrity and functionality of tissues and is modulated in various biological processes. Its visualization and possible quantification using histopathological stains can be important for understanding disease progression or therapeutic response. Visualization of collagen fiber with the histological stain picrosirius red (PSR) is enhanced with polarized light and quantitative analysis is possible using circular polarizers. However, linear polarizers are more commonly available and easier to optically align. The objective of the present study is to demonstrate a novel image acquisition technique and analysis method using linearly polarized light. The proposed imaging technique is based on image acquisition at multiple slide rotation angles, which are co-registered to form a composite image used for quantitative analysis by pixel intensity or pixel counting. The technique was demonstrated on multiple human coronary samples with varying histopathologies and developed specifically to analyze cap collagen in atherosclerotic plaque. Pixel counting image analysis was found to be reproducible across serial tissue sections and across different users and sufficiently sensitive to detect differences in cap structural integrity that are likely relevant to prediction of rupture risk. The benefit of slide rotation angle under linear polarization to acquire images represents a feasible and practical implementation for expanding the general utility of PSR for quantitative analysis.

## Introduction

Fibrillar collagen makes up over 5% of the human body and is the main structural and strength producing component in many organs including tendons, skin, ligaments and the heart [1,2]. In atherosclerosis, extracellular matrix materials, predominantly collagen and elastin, comprise two thirds of the plaque volume in a typical plaque [3,4]. Overproduction of collagen can contribute to plaque growth and luminal stenosis [5]. However, if a plaque contains a necrotic core, a collagen-rich fibrous cap can act as a protective layer of tissue separating the thrombogenic necrotic core from the arterial lumen. Acute coronary events, such as myocardial infarction, are frequently caused by the rupture of unstable atherosclerotic plaques due to a breakdown of fibrillar collagen within the plaque cap, lowering the plaque’s structural integrity, resulting in the release of thrombogenic core materials [6,7]. Methods to measure plaque collagen fiber content are invaluable for both the detection and identification of treatments that can affect collagen in unstable plaques and stenosis. Plaque stabilization with lipid-lowering therapy can restore normal collagen fiber production and reverse the effects of collagen degradation [6,8]. Although intravascular imaging methods such as optical coherence tomography (OCT) can dimensionally evaluate cap thickness, correlation of this type of cap thickness measurement with collagen fiber content remains elusive, particularly with histopathology as the truth reference [9]. Several small studies have shown a lack of correlation between collagen fiber content and cap thickness, further adding to the clinical need to quantify collagen fiber content rather than cap thickness as a measure of plaque stability [10]. In addition to the lack of correlation with existing intravascular imaging technologies, direct histopathology assessment of collagen itself remains mainly qualitative. Given the importance of the role of collagen in atherosclerotic plaque stability, researchers have developed semi-quantitative means for its assessment. The development of an algorithmic tool that combines collagen information with known plaque features, such as plaque and lipid burden, may improve the predictive ability to identify vulnerable plaques and vulnerable patients, thus improving risk stratification [11,12].

A number of methods exist to visualize collagen in histology sections; however, important for studies focusing on fibrillar collagen, it is imperative to be able to use a stain that targets formed collagen fibers, rather than smaller collagen fragments of a specific type such as those targeted by typical antibody stains. Second harmonic generation (SHG) imaging is highly specific to collagen fibers, has a long imaging depth and can be used to image fresh tissue, thereby removing variability from the histological staining process observed with other imaging techniques. SHG has been used routinely for quantitative analysis of collagen particularly fiber alignment and organization [13]. Nonetheless, its use in standard clinical settings is limited due to cost, size and complexity of the instrumentation, and technical expertise requirements [14,15]. On the other hand, picrosirius red (PSR) stain is utilized in many histology labs, at a lower cost and is easier to implement [16]. Although other histological stains can be used to visualize collagen, such as Mason’s trichrome and Movat’s pentachrome, their specificity to collagen is limited and is more likely to fade over time compared to PSR [14,17–19]. Though PSR is not specific to collagen, the stain has a high affinity to collagen from the strong interaction of the dye’s acidic sulfonic groups with the basic groups in collagen. Additionally, PSR is an elongated molecule that aligns itself parallel to the long axis of the collagen fiber which enhances the fiber’s natural birefringence allowing for easier visualization by conventional widefield polarization microscopy [20]. Nonetheless, PSR with polarized light imaging has been mainly used for qualitative assessment of collagen. With commonly utilized linear polarized imaging, some of the collagen fibers can appear dark if aligned with the transmission axis of the polarizers, resulting in the underestimation of collagen [17,18]. To overcome this limitation, collagen can be quantified by imaging the sample with circularly polarized light [17,18]. However, imaging variability with circular polarization can still arise if not properly aligned, which is a common occurrence with many commercially available microscopes [18]. Here we propose a simple technique that minimizes variability with linear polarization by acquiring images at multiple angles of slide rotation to create a co-registered image permitting the optimal visualization of collagen and its quantitative assessment. The proposed method has been shown to be reproducible across serial sections and multiple users. Additionally, the effects of different acquisition parameters, particularly angular rotation and processing threshold, were investigated. The technique was applied to a unique and extensive human coronary study with varying histopathologies and developed specifically to analyze plaque cap collagen within atherosclerosis. Although the fibrous cap is mainly composed of Type I collagen, we expect the technique to be applicable to the assessment of other fibrillar types of collagen, given the known mechanism of PSR bonding to the fibrillary portion of any collagen type.

## Materials and methods

### Ethics approval

Pearl IRB reviewed this study (IRB Study Number: 14-INFR-101) and determined that it was exempt from IRB monitoring due to anonymized donor consent protocols in place by the tissue providers. This review was completed on 9/26/2014, and was granted for the life of the research.

### Histology sample preparation

Human coronary arteries were excised from ten cadaver hearts (International Institute for the Advancement of Medicine, Edison, NJ, National Disease Research Interchange, Philadelophia, PA, and Asterand Bioscence (BioIVT), Detroit, MI). Each heart met at least two criteria for inclusion based on known cardiovascular risk factors (body mass index, and history of either high blood pressure, coronary artery disease, high cholesterol, diabetes mellitus, or smoking). Arteries were mounted in a cage that allowed for accurate tissue sectioning every 2 mm. Starting from the distal end of the artery, each 2 mm slice was removed from the cage and placed with the posterior side, i.e. cut side, up. Two small cuts, at the 6 o’clock and 9 o’clock positions, were placed on the edge of the tissue after sectioning. Tissue marking dye was also used to mark the 10 o’clock position and the proximal side of the tissue. The cuts and the dye serve as markers to help orient tissue slides for microscopy imaging. Each 2 mm slice was fixed in 10% neutral buffer formalin after a maximum of 108 hours post mortem, processed and embedded in paraffin before obtaining a 7 μm thick section that was stained with PSR and mounted on a microscope slide (Mass Histology Service (now Horus Scientific), Worcester, MA).

### Image acquisition

**Fig 1:**
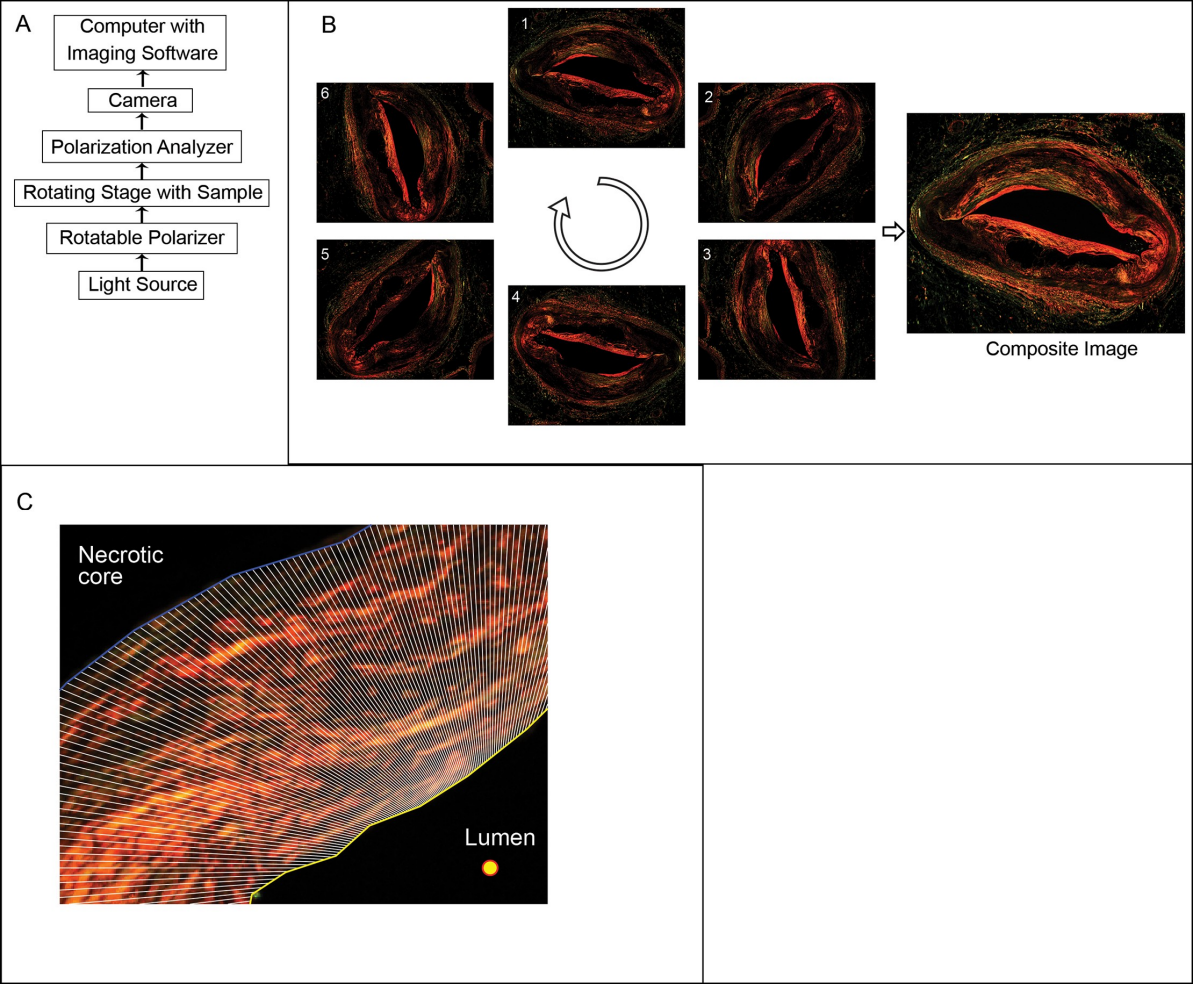
Overview of image acquisition set-up and image processing. (A) Schematic of microscope setup for imaging with linear polarization. (B) Demonstration of image acquisition scheme, where the same microscope slide was imaged at 6 angular rotations (0, 60, 120, 180, 240, and 300 degrees, numbered 1 through 6 above). After acquisition, the 6 images were co-registered and combined into a composite image using the maximum intensity at each pixel location. (C) Higher magnification image showing necrotic core (blue boundary) and the lumen (teal line) bounding the cap region (analysis is restricted to the cap region by this method). Polygons emanating from the lumen center point (yellow/red circle) at one degree intervals (dashed lines) are used for quantification of images for downstream analysis.

The histology images were acquired using a microscope (Olympus BX40), 2x objective lens, and a CCD camera (Q-Imaging RETIGA 1300i FAST, Color 12-bit, 1300x1030 pixels). Images were taken with Q-Capture software (version 1.91.0 with driver QcamDriver DLL version 1.91.10). A 2x objective was selected in order to capture a single image of the entire artery cross-section, from the lumen to the external elastic lamina (EEL) (resolution: 230 pixels/mm or 4.35 μm/pixel). All images were acquired under identical camera settings, including white balance, gain and exposure. The exposure time was selected to limit the occurrence of intensity saturated pixels. A schematic of the microscope setup is shown in Fig 1A.

Each PSR stained slide was placed on a rotatable sample stage, using the marks placed during histology sample preparation to orient the slide. Once the lumen section was centered in the image, the stage was locked in place and the angle of the rotatable stage was noted (this angle was considered to be the starting, or 0° point). One unpolarized image was captured under standard bright field imaging. Without moving the stage or the slide, a polarized image was captured under cross-polarized light, by introducing two linear polarizers in the light path. This automatically co-registered the polarized and unpolarized images at 0°. Cross-polarization of the polarizers was verified, under live image mode, by ensuring maximum black level was achieved. The stage was then rotated counterclockwise by an angular increment (Δϴ). The slide was adjusted laterally, if necessary, to re-center the lumen. This process was repeated, rotating by same angular increment and capturing an image, until the stage was fully rotated 360°. The slide was then removed, and a blank slide was inserted to capture a background image first under polarized light, and then under bright field illumination (no polarizer in the optical path).

### Image processing

All image processing and analysis were performed using MATLAB version 2019b (Mathworks, Natick, MA) and made available in https://dev.azure.com/PSR-Manuscript/PSR%20Manuscript. Polarized PSR images were first background corrected by subtracting the corresponding blank slide image from the histology slide image pixel by pixel. Background corrected and co-registered polarized PSR images were overlaid to form a composite image. For image co-registration, the 0° image was considered the reference image to which subsequent images were registered to. The image to be registered was first converted to grayscale and then rotated according to its associated acquisition angle. This coarse alignment allowed for faster determination of the transformation matrix for final co-registration of the RGB image. This process was repeated for all images for that slide, acquired at different rotation angles. A final composite slide image was formed from the maximum intensity of co-registered images at each pixel position and saved for subsequent image analysis for collagen assessment (Fig 1B).

For image analysis, unpolarized PSR images were used to identify and contour the lumen and necrotic core boundaries. Contours from the unpolarized PSR image were then applied to the corresponding polarized composite image. One degree rays were drawn from a predefined position in the lumen, such as the center, and drawn outwards to the leading necrotic core boundary. Polygons bounded by the rays, lumen, and core were used to define the cap region for collagen assessment (Fig 1C) based on pixel intensity alone or pixel counting with an intensity threshold. The composite image was first converted from RGB to grayscale intensity. For intensity analysis, grayscale pixel intensities were summed or averaged across the cap region. For pixel counting, the number of grayscale pixels above a pre-defined intensity threshold were identified as collagen positive pixels and enumerated. A pixel intensity threshold of 600 counts was selected for image analysis in this study unless otherwise stated. The threshold of 600 counts was determined from the average (300 counts) and standard deviation (232 counts) of intensity in the lumen area, after background correction, from a larger set of 873 similar histology samples for a separate study that utilized the proposed PSR imaging technique to determine collagen deficiency in the cap region and assess the plaque’s vulnerability to rupture.

### Repeatability and responsivity study

Thirty serial sections (7 μm each) were cut from each of four 2 mm tissue blocks. Sections were imaged based on Δϴ = 60° and processed according to methods as described above. All 30 sections were rotated to be co-registered to the first image prior to image analysis based on pixel intensity or pixel counting. Data was reported as a comparison of pixel intensity or pixel counting averaged values across the set of images of interest.

### Reproducibility study

Three different microscope operators were selected to image two slides each. Each operator imaged on a different week, over a span of a month. All operators acquired images with Δϴ = 60° and processed all of the images according to the methods previously described. The pixel counting analyses were performed to determine reproducibility. The percent variation was then calculated as the standard deviation normalized to the average of the total collagen positive pixels in the cap region per operator.

### Angle of rotation study

Six samples of varying atherosclerotic pathology, from different hearts and artery segments, were selected to study the effect of varying Δϴ. Two of the sections were identified as thin-capped fibroatheroma (TCFA) with minimum cap thickness < 100 microns, specifically 42 and 72 μm. The remaining samples are thick capped, with minimum cap thickness ranging from 120 to 275 μm. Images were acquired as above for different Δϴ, specifically at 2°,6°, 12°, 44° and 60°. Considering fiber alignment is symmetric over a total rotation of 180°, the selected angles approximately divide 180° equally.

The same set of images captured for each Δϴ were additionally used to create composite images using only half the data set, corresponding to a 180° range of rotation as opposed to 358° (ϴ_R,full_). Specifically, images captured over 0 to 178° (ϴ_R_, _low_) were combined for one composite image, while those captured over 180° to 358° (ϴ_R_._high_) were combined for a comparative composite image. The number of composite images formed are summarized in Table 1.

**Table 1:** Summary of composite images and associated Δϴ and range of rotation angle (ϴ_R,full_ = 0° to 358°, ϴ_R,low_ = 0°to 178° and ϴ_R,high_ = 180°to 358°) for a single histology slide.

Composite Image#	Δϴ [degrees]	Range of Rotation [degrees]	Composite Image#	Δϴ [degrees]	Range of Rotation [degrees]	Composite Image#	Δϴ [degrees]	Range of Rotation [degrees]
1	2	ϴ_R,full_	6	2	ϴ_R,low_	11	2	ϴ_R,high_
2	6	ϴ_R,full_	7	6	ϴ_R,low_	12	6	ϴ_R, high_
3	12	ϴ_R,full_	8	12	ϴ_R,low_	13	12	ϴ_R, high_
4	44	ϴ_R,full_	9	44	ϴ_R,low_	14	44	ϴ_R, high_
5	60	ϴ_R,full_	10	60	ϴ_R,low_	15	60	ϴ_R, high_

### Pixel intensity threshold study

Additional analysis was performed to determine the effect of selected pixel intensity threshold for pixel counting. Pixel counting analysis was performed for threshold intensity ranging from 0 to 1,000 counts.

## Results

### Repeatability and responsivity

**Fig 2:**
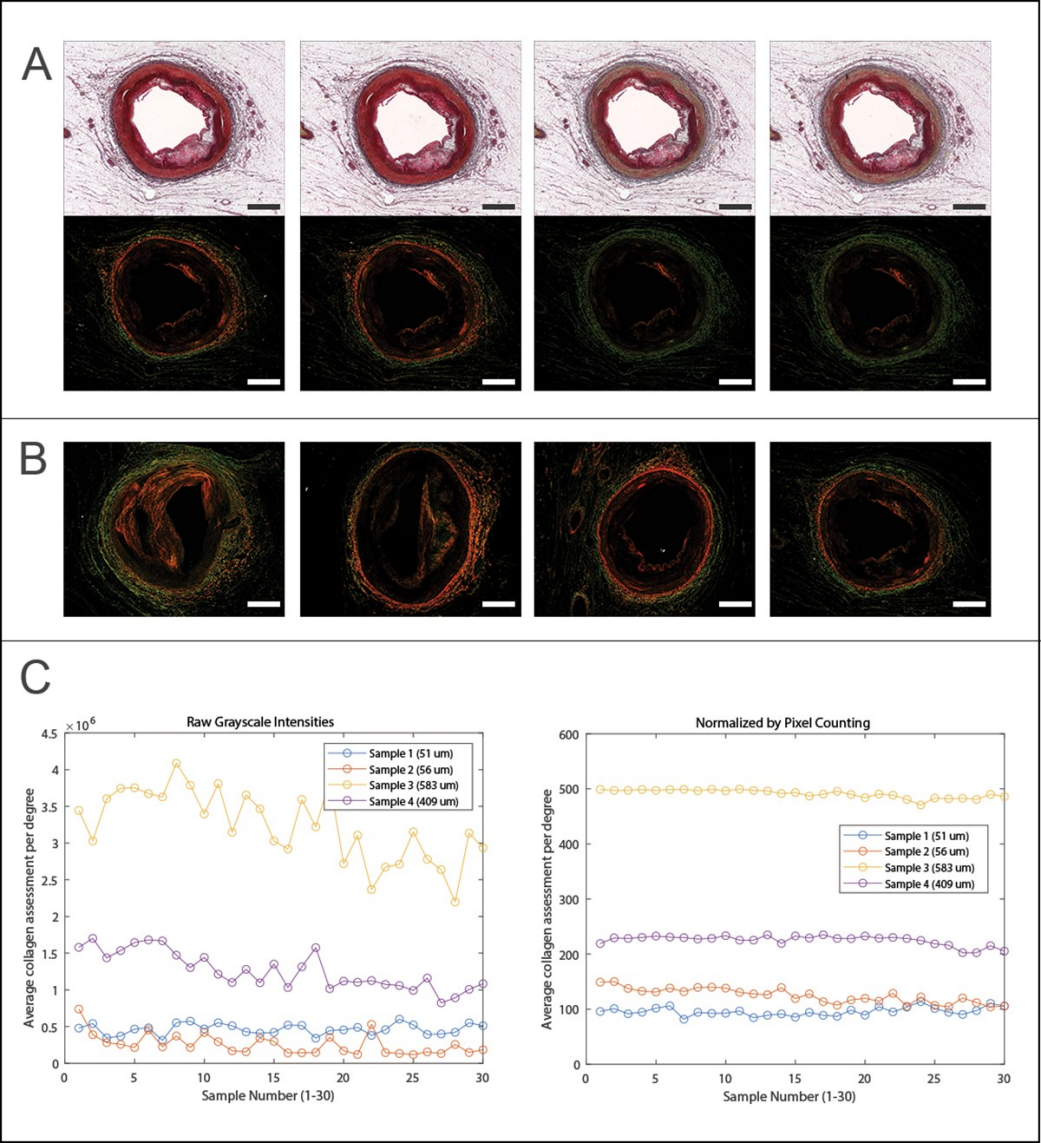
Repeatability. (A) Four example serial sections (each cut at 7 μm thick) from the same 2 mm tissue block, and changes to both bright light (top) and polarized light (bottom) images are observed in the cap region. (B) Four different sections (numbered from left to right) from 3 different hearts showed increased repeatability in (C) with pixel counting method compared to use of grayscale intensity. Scale bars = 1 mm.

Results across different pathologies showing similar levels of variability in color and intensity were observed across the thirty serial sections with some sections showing more orange pixels, and other showing more green pixels, as shown in Fig 2A and 2B. These RGB variations manifest as intensity variations in grayscale color. Average grayscale intensity values per degree show variability in the collagen assessment method. In comparison, the pixel counting method shows a much flatter response or consistent collagen positive pixel count for the slide regardless of the pathology or predominant color observed in the actual images, as shown in Fig 2C.

**Fig 3:**
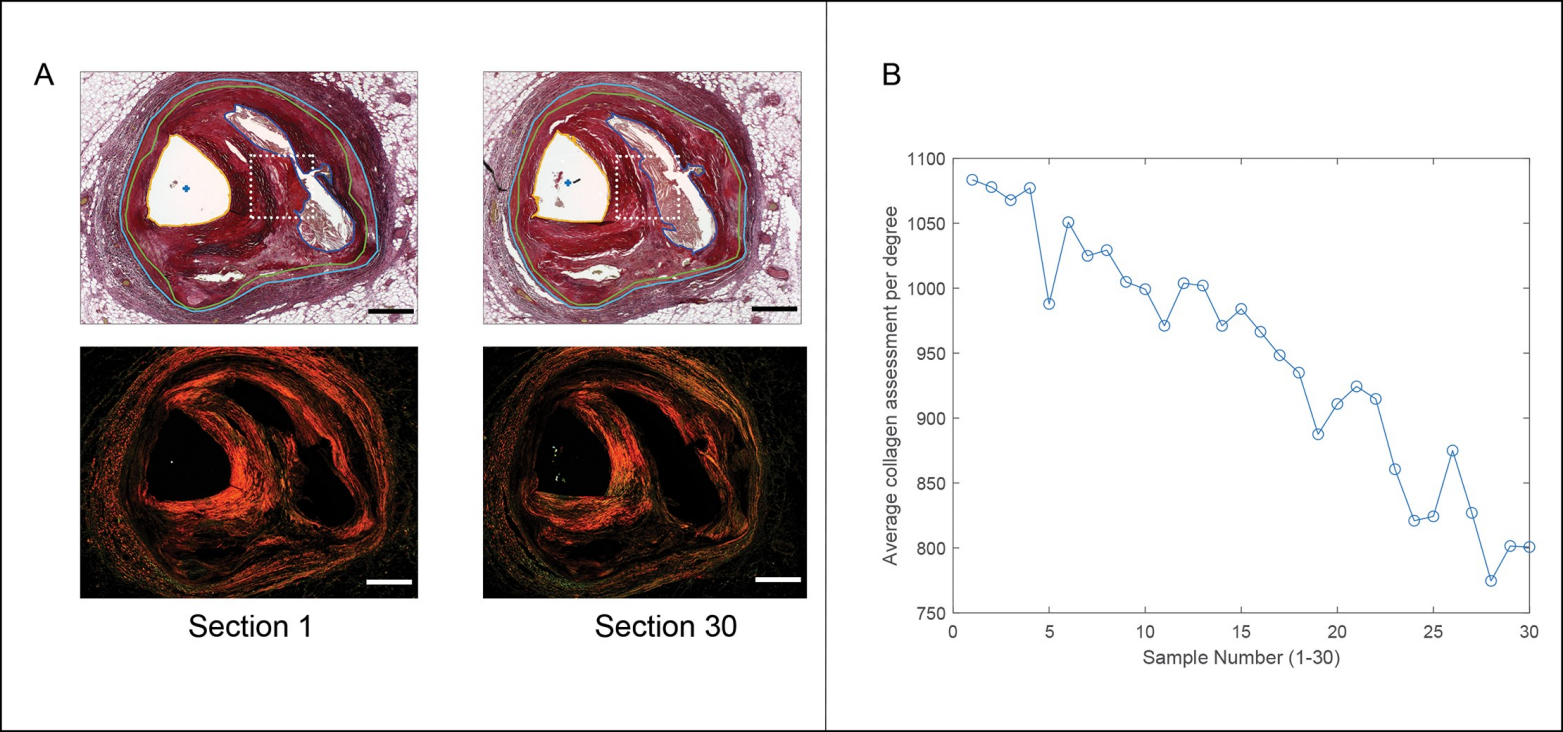
Responsivity of pixel counting to changes in cap thickness. (A) First and last of thirty serial sections (each cut at 7 μm thick), illustrating changing thickness of necrotic core and cap thickness at mid region of necrotic core arc (dotted white box), bright light images (top) and polarized images (bottom). Scale bars = 1 mm. (B) Plot of pixel counting method illustrating decreasing magnitude of positive cap pixels throughout the sections as the cap thickness decreases.

The responsivity of the pixel counting technique with changing pathology was assessed from one of the samples, Fig 3. A thick-capped fibroatheroma sample was observed where the cap thickness changed substantially across the length of the 30 sections (approximately 210 microns from first to last section within the 2 mm tissue block). In this sample, the ability of the pixel counting method to capture this change was apparent, with a maximum cap thickness of 1117 microns and approximately 1100 average collagen positive pixels in the first section, and a maximum cap thickness of 701 microns and approximately 800 average collagen positive pixels in the last section.

### Reproducibility

Applying the standard pixel counting method to the three user’s images, resulted in an average of 95% agreement. This percentage is the average percent of pixels that are the same, across all three users, relative to the average number of pixels within the cap region. This high average percentage shows that there is only a small difference in pixels within the cap across images, demonstrating that the image acquisition of the two slides was reproducible across users and time.

### Angle of rotation

**Fig 4:**
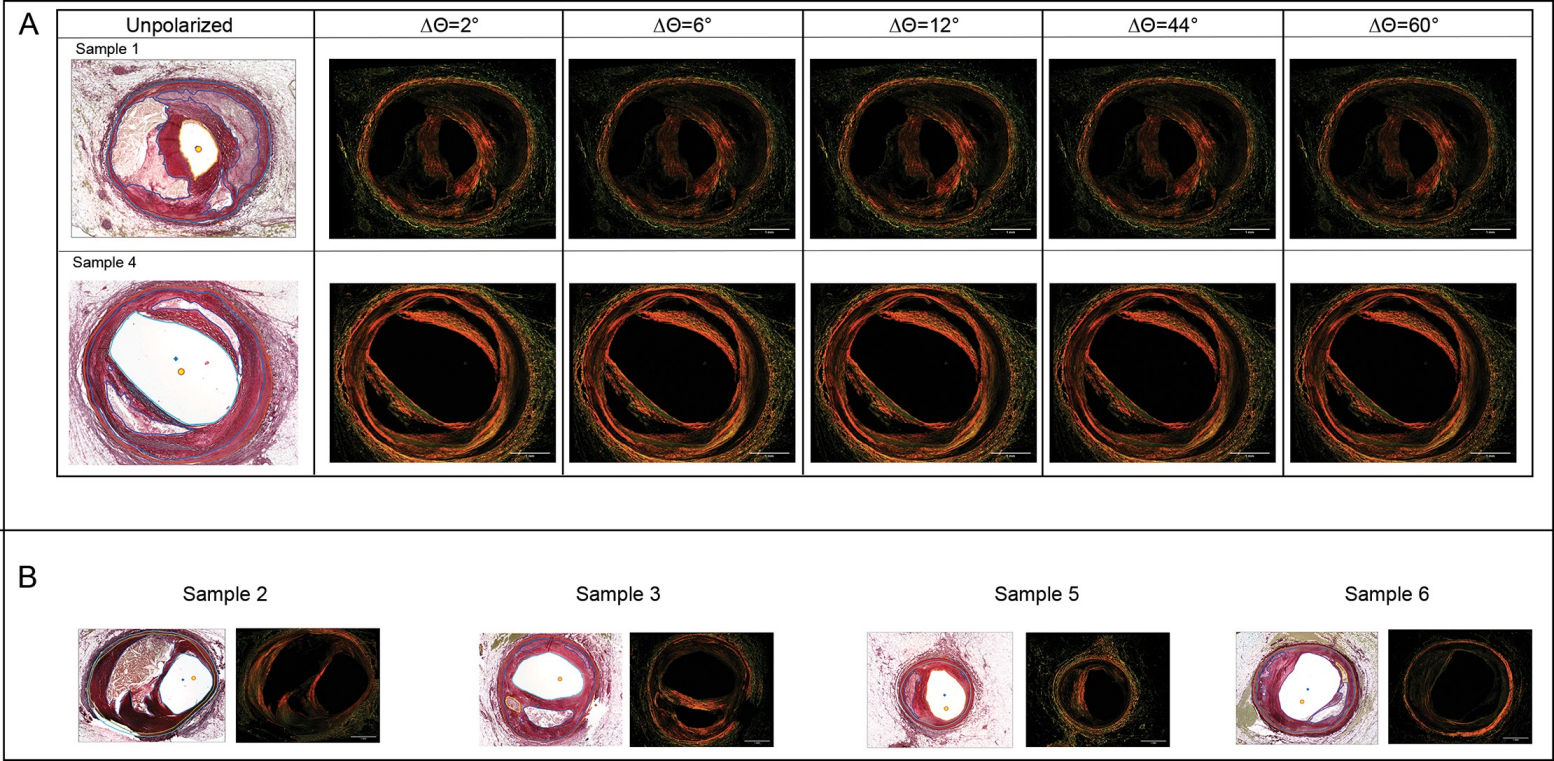
Unpolarized image and corresponding polarized co-registered composite images from different Δϴ. (A) First row and second rows correspond to an example of a section with a thick collagen cap (124μm) and thin cap (43μm), respectively. (B) Unpolarized and corresponding polarized composite images (Δϴ = 60°) of other samples for angle of rotation analysis. Scale bars = 1 mm.

The effect of slide rotation angular increment, Δϴ, on the assessment of collagen was determined for both grayscale intensity-based, and pixel counting methods. The total gray scale intensity and total pixel count over the cap region for each sample were quantified from composite images formed for Δϴ = 2°, 6°, 12°, 44°, and normalized to those from Δϴ = 60° for comparison. Co-registration of composite images to the corresponding composite image from Δϴ = 60°, as shown in Fig 4, ensures analysis and calculations were derived from the same angular position and polygon.

Results, summarized in Tables 2 and 3, show larger variation in intensity as compared to pixel counting as the Δϴ is reduced. Composite images formed from Δϴ = 2° had 25–30% higher intensity but only <3% more pixel counts compared to composite images from Δϴ = 60°.

**Table 2:** Comparative results of total pixel intensity in the cap region for different Δϴ.

Sample#	Δϴ = 2/Δϴ = 60	Δϴ = 6/Δϴ = 60	Δϴ = 12/Δϴ = 60	Δϴ = 44/Δϴ = 60
1	1.30	1.23	1.17	1.02
2	1.26	1.20	1.14	0.99
3	1.27	1.21	1.16	1.02
4	1.27	1.20	1.16	1.01
5	1.28	1.21	1.16	1.02
6	1.33	1.25	1.19	1.03

**Table 3:** Comparative results of total pixel counts in the cap region for different Δϴ.

Sample#	Δϴ = 2/Δϴ = 60	Δϴ = 6/Δϴ = 60	Δϴ = 12/Δϴ = 60	Δϴ = 44/Δϴ = 60
1	1.00	1.00	1.00	1.00
2	1.01	1.01	1.01	1.01
3	1.01	1.01	1.01	1.01
4	1.01	1.01	1.00	1.00
5	1.00	1.00	1.00	1.00
6	1.02	1.02	1.02	1.00

As the slide is rotated, the orientation of the collagen fiber relative to the polarization axis of the linear polarizers will be similar after rotating 180 deg. A collagen fiber oriented 45° relative to the horizontal plane at slide rotation = 6° will be oriented similarly at slide rotation = 186°. Calculated total intensity and total pixel counts for composite images from images acquired from slide rotation 0° to 178° (ϴ_R_,_low_) and 180° to 358°(ϴ_R_,_high_) are expected to be similar compared to values from corresponding composite image from images acquired over slide rotation 0° to 358° (ϴ_R_,_full_).

Results, summarized in Tables 4 and 5, show that normalized pixel intensities are typically lower and more variable compared to normalized pixel counts. Normalized pixel intensity average and standard deviation are 0.931 and 0.026, respectively, compared to 0.997 and 0.003 from normalized pixel counts. Additionally, larger differences can also be observed in normalized pixel intensity between ϴ_R_,_low_ and ϴ_R,high_.

**Table 4:** Results of total intensity in the cap region from composite images from half of the range of rotation angles compared to the full range.

Sample#	ϴ_R,low_/ϴ_R,full_					ϴ_R,high_/ϴ_R,full_				
	Δϴ = 2	Δϴ = 6	Δϴ = 12	Δϴ = 44	Δϴ = 60	Δϴ = 2	Δϴ = 6	Δϴ = 12	Δϴ = 44	Δϴ = 60
1	0.960	0.955	0.954	0.938	0.925	0.920	0.915	0.908	0.898	0.890
2	0.960	0.958	0.958	0.943	0.928	0.910	0.902	0.904	0.883	0.899
3	0.956	0.954	0.954	0.955	0.951	0.913	0.907	0.897	0.916	0.870
4	0.944	0.943	0.939	0.933	0.942	0.948	0.938	0.933	0.930	0.893
5	0.939	0.931	0.923	0.935	0.893	0.942	0.941	0.937	0.931	0.919
6	0.929	0.922	0.915	0.919	0.910	0.916	0.912	0.910	0.895	0.879

**Table 5:** Results of total pixel counts in the cap region from composite images from half of the range of rotation angles compared to the full range.

Sample#	ϴ_R,low_/ϴ_R,full_					ϴ_R,high_/ϴ_R,full_				
	Δϴ = 2	Δϴ = 6	Δϴ = 12	Δϴ = 44	Δϴ = 60	Δϴ = 2	Δϴ = 6	Δϴ = 12	Δϴ = 44	Δϴ = 60
1	0.999	0.999	0.999	0.998	0.998	0.999	0.999	0.999	0.998	0.997
2	0.998	0.998	0.998	0.996	1.001	0.998	0.996	0.996	0.995	0.998
3	0.999	0.999	0.999	0.998	1.001	0.997	0.997	0.995	0.996	0.995
4	0.998	0.997	0.997	0.995	0.994	0.999	0.998	0.998	0.999	0.996
5	1.000	1.000	1.000	0.999	0.999	0.999	0.999	0.999	0.999	0.999
6	0.990	0.983	0.975	0.969	0.954	0.981	0.974	0.965	0.935	0.926

### Pixel intensity threshold

**Fig 5:**
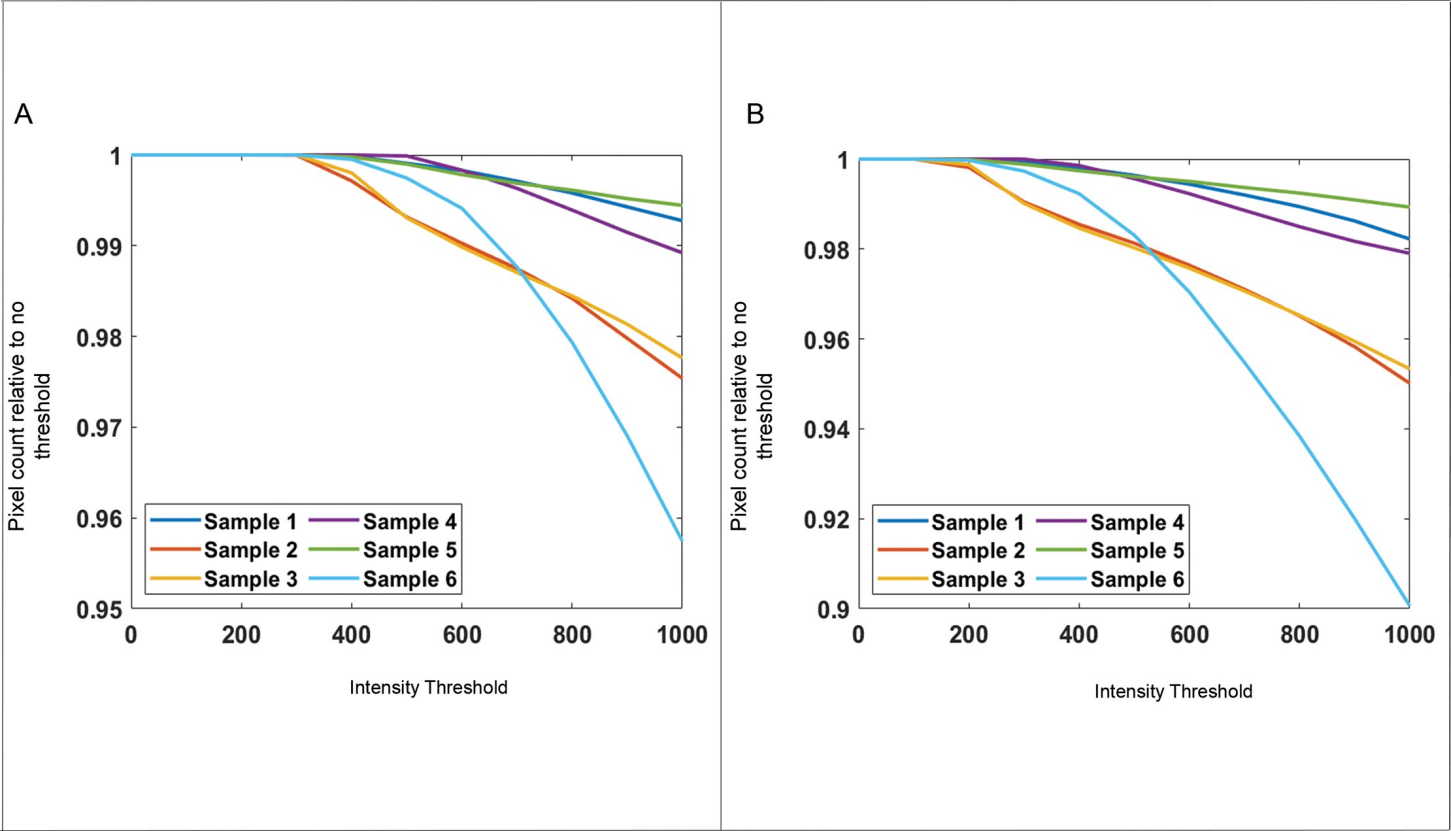
Total number of collagen positive pixels in cap region for different threshold for pixel counting. Values shown are relative to no threshold (intensity value = 0). (A) Composite image from Δϴ = 2° and ϴ_R,full_ and (B) Composite image from Δϴ = 60° and ϴ_R,full_.

The effect of pixel intensity threshold used for pixel counting was assessed. Fig 5 displays the trend for the smallest and largest Δϴ. As expected, the number of pixels determined as collagen positive decreased with increasing pixel intensity threshold since dimmer pixels are removed for enumeration. At the typically used intensity threshold of 600 counts, the number of collagen positive pixels is > 98% of collagen positive pixels with no intensity threshold.

## Discussion

Reproducible and responsive collagen assessment of fibrous cap with linearly polarized imaging is feasible as shown on atherosclerotic plaque samples. Whittaker et al. proposed the use of circular polarization to quantify fibrillar collagen to address the shortcomings of linear polarization, namely the orientation dependency of the fiber relative to the linear polarizer’s transmission axis, affecting the ability to visualize collagen [17]. Alternatively, they proposed changing the orientation of the tissue section with the use of a rotating microscope to minimize this orientation mediated issue; however, while suggested, this approach was not tested. The motivation of our study is to develop a reproducible fibrillar collagen quantification technique based on images acquired as the sample was rotated under linearly polarized light, from which a composite image was created and pixel counting analysis was performed.

Repeatability and reproducibility of our technique was demonstrated by assessing the variation introduced from histological processing and from different imaging personnel. It is known that colors in histology slides can vary due to inconsistencies, such as in the sample thickness and staining, despite the use of the same chemical dye [21]. For our repeatability study, an attempt was made to reduce variation from the staining procedure by batch staining the serial sections from the same tissue block. Nonetheless, color and intensity variation between serial sections were observed. Colors of collagen fibers under polarized light have been thought to depend on fiber type, collagen packing, or fiber/sample thickness [18,22–25]. However, within adjacent tissue slices, the fiber type or collagen packing is expected to be similar. The observed variation is likely attributed to slice thickness variation or related artifacts from tissue sectioning. Given the visual variations observed due to small changes in sample thickness, our study points to a practical challenge in collagen quantification with PSR. Thicker tissue slices or tighter tolerance in tissue slicing may minimize the issue; however, it will be difficult to completely remove this type of sample variability, due to the manual nature of tissue slicing. Alternatively, we found that pixel counting image analysis is insensitive to variations in tissue slices as shown by excellent repeatability across different atherosclerotic plaque morphologies and stages of disease. Additionally, pixel counting has been shown to produce reproducible results across multiple imaging personnel over different days. At the same time, the method has been shown to maintain responsivity to capture changes in collagen content. Reproducibility and responsivity are necessary hallmarks of useful stains and imaging methods, particularly for studies that require large number of samples or where precise quantification is necessary, such as in discriminating stages of disease progression or tracking morphological changes in response to therapy.

Linear polarization with sample rotation may still underestimate the total amount of fibrillar collagen, particularly if the tissue sample contains a number of wavy or crimped fibers [18]. This concern was addressed by obtaining multiple images over the sample rotation, at a given angular increment and performing image analysis on the composite image instead of individual images. Intensity analysis of the composite images showed that the smallest angular increment, i.e. 2°, is associated with the highest total pixel intensity in the cap region and the largest angular increment, i.e. 60°, is associated with the lowest pixel intensity. However, pixel counting shows <3% relative difference in collagen pixel positive count between the smallest and largest angular increment. This indicates that despite the variation in total intensity, there is sufficient intensity in all of the composite images for dim collagen fibers to be assigned as a collagen positive pixel and be quantified. This similarly explains the observed smaller variation with pixel counting on composite images formed over a half or over the full range of rotation. Given that the slides are manually rotated and sometimes require re-centering depending on the sample morphology, the angle that an image is acquired will likely be inexact. Small differences in the slide rotation angle, and hence relative alignment of the collagen fibers to the polarizer axis, provides a possible explanation for the large difference in relative total intensity between composite images from slide rotation range 0° to 178° and 180° to 358°. Though a motorized stage can minimize this, error in the angle may be expected from motor back-lash or hysteresis, and morphological effects requiring re-centering would still be present. Nonetheless pixel counting is less sensitive, compared to pixel intensity analysis from errors in the angle during image acquisition. Results from the current study indicate that practical implementation of linearly polarized imaging of PSR technique is feasible for collagen quantification, and sufficient for most quantitative analyses. At the expense of <3% collagen positive pixels counted, the total number of images to create a composite image with an angular increment of 60° instead of 2° is reduced from 180 to 6. Assuming 20 seconds is needed to rotate and acquire an image, the total time to acquire slide images to create the composite image is reduced from 60 to 2 minutes. At an additional expense of <2% collagen positive pixels counted by only acquiring images over half of the range of rotation, the total time needed to acquire a single slide can be further reduced to 1 minute.

The point counting and digital analysis for collagen quantification described by Whittaker is similar to the pixel counting analysis described here [17,26]. Rich and Whittaker later extended the image analysis from gray scale to color images [18]. Background subtraction was performed after CYMK color separation to remove interstitial space and non-collagen elements. The brightness of the background prior to image subtraction was adjustable by the user to ensure that the thinnest fibers were still visible. However, the ability of the user to adjust image parameters may result in user to user variability in the final collagen assessment. Conversely, with our technique, no post background subtraction was performed. It is feasible that collagen fiber pixels, especially from thin fibers, were removed prior to forming the composite image. However, we expect this effect to be minimal mainly since images in our study were acquired at a low magnification, 2x, to image the entire cross section of the artery without the need to concatenate multiple images by image stitching. Additionally, the blank slide for background subtraction was obtained under the same cross-polarized illumination as the sample slide for each sample imaged. Rich and Whittaker also exploited color to assess the proportion of different fiber thicknesses by hue. Our image analysis is simply performed on grayscale images, similar to the image analysis originally proposed by Whittaker, since we only deemed it necessary to assess all fibrillar collagen in the cap, rather than attempting to separate by color or fiber thickness [27]. Additionally, our analysis on within-sample variability, indicates that the fiber color is highly influenced by the thickness of the tissue slice. The reproducibility or interpretation of color-based analysis from serial sections should be performed to determine if the variations in color are from changes in the collagen structure from biological mechanisms (such as wound healing or disease progression) or from how the tissue was processed for histology. Regardless of image analysis technique used, one shortcoming of our study is the lack of comparison to circular polarization. A potential future study is one that compares PSR stained sections imaged with circularly and linearly polarized light using the same image analysis technique against a gold standard for fibrillar collagen concentration, such as a collagen specific assay.

As mentioned, a second limitations of our technique is the possibility that weakly birefringent fibers were lost following background subtraction. It may be feasible that selection of intensity threshold can minimize this issue but will need to be explored in future studies. Additionally, no image assessment was performed to ensure quality of background subtracted images to assess for issues such as the presence of contaminants on the blank slide that appear bright under polarized light or that the cross-polarized illumination has not changed from accidental movements of either polarizers. The latter can be addressed by comparing a blank section of the slide visible on all individually acquired images, such as the lumen, prior to composite image formation. Bright contaminants on the blank slide are only a concern if they are found in the region of interest, e.g. plaque cap. Additional studies can compare pixel counts on composite images with and without background correction. Future studies can also explore the performance of pixel counting analysis on images acquired at higher magnification. An expected challenge of our method is the sheer number of images required to be stitched to create a composite image for samples requiring a large field of view at high magnification. A method to organize the images and minimize human error during the many acquisition steps would be useful. Finally, all image processing and analysis were performed using MATLAB. Though some of the image processing, such as background subtraction and image overlay are available in some software packages such as ImageJ, a number of the quantification and image analysis scripts were custom made in MATLAB to automate some of the processing. One example is a program that iteratively checks the formed composite image against quality metrics, and performs image transformation to improve the co-registration, if necessary.

PSR, in conjunction with polarized imaging, is a simple and commonly used histology stain that allows for the visualization of collagen fibers. Quantitative analysis with PSR stain has been demonstrated with circular polarization. However, circular polarizers are not standard or readily available for all optical configurations, and care must be taken to ensure proper optical alignment. Our study provides an alternative quantitative technique that uses commonly found linear polarized imaging for the general goal of further promoting the utility of PSR for quantitative analysis of fibrillar collagen in histological samples.
